# Efficacy of Proanthocyanidins from *Pelargonium sidoides* Root Extract in Reducing *P. gingivalis* Viability While Preserving Oral Commensal *S. salivarius*

**DOI:** 10.3390/ma11091499

**Published:** 2018-08-22

**Authors:** Nijole Savickiene, Aiste Jekabsone, Lina Raudone, Asmaa S. Abdelgeliel, Andrea Cochis, Lia Rimondini, Elina Makarova, Solveiga Grinberga, Osvalds Pugovics, Maija Dambrova, Ingrida M. Pacauskiene, Nomeda Basevičiene, Pranas Viškelis

**Affiliations:** 1 Department of Pharmacognosy, Lithuanian University of Health Sciences, Sukileliu av. 13, LT-50161 Kaunas, Lithuania; lina.raudone@lsmuni.lt; 2 Laboratory of Pharmaceutical Sciences, Institute of Pharmaceutical Technologies, Lithuanian University of Health Sciences, Sukileliu av. 13, LT-50161 Kaunas, Lithuania; aiste.jekabsone@lsmuni.lt; 3 Laboratory of Molecular Neurobiology, Neuroscience Institute, Lithuanian University of Health Sciences, Eiveniu str. 4, LT-50161 Kaunas, Lithuania; 4 Department of Health Sciences, University Piemonte Orientale, Via Solaroli 17, 28100 Novara, Italy; asmaa.elgafari@sci.svu.edu.eg (A.S.A.); andrea.cochis@med.uniupo.it (A.C.); lia.rimondini@med.uniupo.it (L.R.); 5 Department of Botany, South Valley University, Qena 83523, Egypt; 6 Latvian Institute of Organic Synthesis, Aizkraukles str. 21, LV1006 Riga, Latvia; elina.makarova@farm.osi.lv (E.M.); solveiga@osi.lv (S.G.); osvalds@osi.lv (O.P.); maija.dambrova@farm.osi.lv (M.D.); 7 Clinic of Dental and Oral Pathology, LSMU Hospital, Kaunas Clinics, Medical Academy, Lithuanian University of Health Sciences, Eivenių str. 2, LT-50161 Kaunas, Lithuania; ipacauskiene@gmail.com (I.M.P.); bnomeda@gmail.com (N.B.); 8 Laboratory of Biochemistry and Technology, Institute for Horticulture, Lithuanian Research Centre for Agriculture and Forestry, Kauno str. 30, LT-54333 Babtai, Lithuania; biochem@lsdi.lt

**Keywords:** *Pelargonium sidoides* root extract, proanthocyanidins, LC/MS/MS, antioxidant activity, bacteriotoxicity

## Abstract

Bacterial resistance to antibiotics and the disruption of beneficial microbiota are key problems in contemporary medicine and make the search for new, more efficient infection treatment strategies among the most important tasks in medicine. Multicomponent plant-derived preparations with mild antibacterial activity created by many simultaneous mechanisms together with anti-inflammatory, innate immune and regenerative capacity-stimulating properties are good candidates for this therapy, and proanthocyanidins are among the most promising compounds of this sort. In this study, we have isolated proanthocyanidins from *Pelargonium sidoides* DC root extract and characterized and compared the composition, antioxidant properties and antibacterial activity of the proanthocyanidin fraction with those of the whole extract. The results revealed that proanthocyanidins had significantly stronger antioxidant capacity compared to the root extract and exhibited a unique antibacterial action profile that selectively targets Gram-negative keystone periodontal and peri-implant pathogenic strains, such as *Porphyromonas gingivalis*, while preserving the viability of beneficial oral commensal *Streptococcus salivarius*. The finding suggests that proanthocyanidins from *Pelargonium sidoides* root extract are good candidates for the prolonged and harmless treatment of infectious diseases.

## 1. Introduction

Increasing antibiotic resistance is one of the most important global health problems today and is making infection treatments increasingly challenging and leading to longer hospital stays, higher medical costs and increased mortality [1]. Another problem related to the administration of antibiotic drugs and the application of bactericidal chemicals is that they destroy not only pathogens but also commensal strains of human microbiota which are important partners that preserve homeostasis in the skin and mucosal physiological systems of the human body [2,3,4]. Multicomponent herbal antibacterial medicines are a promising source of new therapeutic agents for treatment that do not pose the risk of resistance development [5,6]. The diversity of antibacterial components in phytopreparations also places them among promising candidates for pathogen-selective activity testing. African geranium, or *Pelargonium sidoides* DC (PS), is an indigenous medicinal plant of South Africa that has been widely used as a traditional medicine for over a century [7]. Various preparations of this plant, and in particular the root extracts, have demonstrated multiple antibacterial and immune system stimulating properties [8,9,10]. PS root extract (PSRE) was effective in clinical trials for the treatment of various respiratory system infections [11,12,13], and the properties of the material suggest the area of application may be extended to a much greater range of infection cases. As a multicomponent preparation with a range of synergistically acting antibacterial and other biological activities, PSRE administration is not accompanied by the risk of the evolvement of treatment-resistant pathogenic strains. This suggests that PSRE may be applicable for prolonged and repeated usage and, therefore, a good candidate as an antibiotic and chemical bactericide therapy substitute and/or additive in cases of chronic and repeated infections. However, the antibacterial effects of PSRE are rather mild and might be not sufficiently effective if applied for the treatment of severe infection. The antibacterial activity of PSRE is mostly attributed to proanthocyanidins (PACN) which are also reported to possess antioxidant, anti-inflammatory, anti-aging and anticancer properties [14,15,16]. PACN-enriched extracts from other herbal sources inhibit biofilm formation and the adhesion of periodontopathogenic bacteria and suppress bacterial proteolytic activity, cytokine and MMP (matrix metalloproteinases) production by immune and mucosal cells [16,17,18]. Taking this into account, it is likely that PACN isolated from PSRE and concentrated has stronger antibacterial activity yet preserves the same advantage of no risk of resistance because of its multiple action pathways. To test this hypothesis, we have isolated a PACN fraction from PSRE, analysed its composition and compared its antibacterial efficiency with that of PSRE. To investigate the selectivity of antimicrobial activity, two bacterial strains were selected to assay PSRE and PACN antibacterial efficacy: (i) The Gram-negative biofilm former anaerobic keystone pathogen *Porphyromonas gingivalis*, a well-known player in the development of periodontal disease and peri-implantitis [19,20,21,22]; and (ii) the Gram-positive aerobic oral commensal *Streptococcus salivarius*.

## 2. Materials and Methods

### 2.1. Chemicals and Solvents

All reagents used for the study were of analytical and chromatographic grade. Acetonitrile, methanol, (+)-catechin and (−)-epicatechin, -hydroxy-2,5,7,8-tetramethylchroman-2-carboxylic acid (Trolox), 2,2-azinobis (ethyl-2,3-dihydrobenzothiazoline-6-sulphonic acid) diammonium salt (ABTS), potassium persulphate were purchased Fluka, epigallocatechin, epigallocatechin gallate, gallic acid, quercetin, delphinidin, cyanidin, 1-butanol, Sephadex LH-20, hydrochloric acid, acetone, ferric ammonium sulphate, phosphoric acid, 85 wt %, sodium acetate trihydrate, iron(III) chloride hexahydrate, and 2,4,6-tripyridyl-s-triazine (TPTZ) from Sigma-Aldrich (Steinheim, Germany), 99.8% trifluoracetic acid from Carl Roth (Karlsruhe, Germany), 96% ethanol from Vilniaus degtine AB (Vilnius, Lithuania). The purified deionized water (18.2 mΩ/cm) was produced using the Millipore water purification system. The *Pelargonium sidoides* root extract (PSRE) was purchased from UNILAB, LP (Rockville, MD, USA) (extraction medium 50% methanol).

### 2.2. Purification of PACN

Purification of PACN was performed as described by [23]. Briefly, 4 g of PSRE was dissolved in 200 mL of 50% methanol, and the solution was centrifuged at 2000× *g* for 20 min and filtered through 0.45 µm nylon filters (Roth GmbH, Karlsruhe, Germany). The solution was purified by gel adsorption over Sephadex LH-20. The PACN were released from the gel with 70% aqueous acetone (500 mL) and concentrated under vacuum at 35 °C. The aqueous aliquot was freeze-dried.

#### Acid/n-Butanol Hydrolysis of Extracts

PACN extract was weighed to 0.05 g (accurate sample), placed into a conical flask with 50 mL of 50% methanol, vortexed for 2 min and filtered through 0.22 µm filters and subjected to analysis. PACN were hydrolysed according to the method described by Porter et al. [24]. Briefly, 6 mL of the n-butanol/HCl reagent (950 mL of n-butanol and 50 mL concentrated HCl), 1.0 mL of the extract, and 0.2 mL of the iron reagent were added. The flask was connected under reflux in a boiling water bath for 60 min. Then, the solution was cooled and transferred to a volumetric flask and adjusted to 25 mL with the n-butanol/HCl reagent.

### 2.3. Qualitative and Quantitative Analysis

#### 2.3.1. HPLC Analysis

Chromatographic analysis was carried out using a Waters Alliance e2695 Separations Module equipped with a Waters 2998 PDA Detector (Milford, CT, USA).

For the HPLC analysis of extracts, an accurate sample (0.05 g) was placed into a conical flask with 50 mL of 50% methanol, vortexed for 2 min and then filtered through 0.22 µm filters and subjected to analysis. The separation was performed on an ACE Excel 3 SuperC18 analytical column (Aberdeen, Scotland) (250 × 4.6 mm, 3 μm) at 25 °C. The mobile phase consisted of 0.1% TFA in deionized water (A) and acetonitrile (B). The gradient elution was as follows: 0–30 min, 15–30% B; 30–50 min, 30–60% B; 50–55 min, 60–90% B; and 55–60 min, 90–15% B. The flow rate was 0.5 mL/min, and the injection volume was 10 μL. The detector was set in the 200–400 nm range. The chromatographic data were acquired and processed with Empower 3 software (Milford, CT, USA).

For the HPLC analysis of extracts after butanol hydrolysis, the separation was performed on an ACE Excel 5 SuperC18 analytical column (Aberdeen, Scotland) (250 × 4.6 mm, 5 μm) at 25 °C. The mobile phase consisted of 4% Phosphoric acid in deionized water (A) and acetonitrile (B). The gradient elution was as follows: 0–10 min, 15–30% B; 10–15 min, 30–90% B; 15–17 min, 90–90% B; 17–18 min, 90–15% B; and 18–25 min, 15% B. The flow rate was 1 mL/min, and the injection volume was 10 μL. The detector was set at the 550 nm. The chromatographic data were acquired and processed with Empower 3 software (Milford, CT, USA).

Chromatographic peak identification was carried out according to the analyte and reference compound retention time, as well as by comparing the UV absorption spectra of the reference compounds and analytes obtained with a diode array detector.

#### 2.3.2. UPLC-ESI-MS Conditions

Phenolic compounds were separated using an Acquity H-class UPLC system (Waters, Milford, CT, USA) equipped with a Xevo triple quadrupole tandem mass spectrometer (Waters, Milford, CT, USA). An electrospray ionization (ESI) source was used to obtain MS and MS/MS data. An Acquity BEH C18 column (50 × 2.1 mm, 1.7 μm) (Waters, Milford, CT, USA) was used for analysis. The column temperature was maintained at 40 °C. Gradient elution was performed with a mobile phase consisting of 0.1% water solution of formic acid (solvent A) and acetonitrile (solvent B), with the flow rate set to 0.5 mL/min. A linear gradient profile was applied with the following proportions of solvent A: initial—95%, 1 min—80%, 7.5 min—75%, 8 min—0%, 10 min—95%. Negative electrospray ionization was applied with the following settings: capillary voltage—2.5 kV, source temperature—150 °C, desolvation temperature—500 °C, desolvation gas flow—800 L/h, cone gas flow—20 L/h. MS spectra were recorded in the range of 80–2000 *m*/*z*.

#### 2.3.3. UPLC-MS/MS Conditions

Sample analysis for the determination of the mean degree of polymerization was carried out on an Acquity UPLC system (Waters, Milford, CT, USA) equipped with a Quattro micro triple quadrupole tandem mass spectrometer (Waters, Milford, CT, USA). An electrospray ionization (ESI) source in negative mode was used to obtain MS/MS data. An Acquity HSS T3 column (50 mm × 2.1 mm, 1.8 μm) (Waters, Milford, CT, USA) was used for analysis. The column temperature was maintained at 30 °C. Gradient elution was performed with a mobile phase consisting of 0.1% water solution of formic acid (solvent A) and acetonitrile (solvent B), with the flow rate set to 0.25 mL/min. A linear gradient profile was applied with the following proportions of solvent A: initial—95%, 0.5 min—2%, 4.5 min—2%, 4.7 min—95%, 6 min—95%. Negative electrospray ionization was applied with the following settings: capillary voltage—3.0 kV, source temperature—150 °C, desolvation temperature—400 °C, desolvation gas flow—800 L/h. For the detection of procyanidin and prodelphinidin terminal and extension units following multiple reaction monitoring (MRM) transitions 287.1→125.4, 289.1→245.4, 303.2→125.1, 305.2→125.1 at five cone voltage energy values (50, 75, 85, 110 and 140 V for procyanidins and 55, 80, 110, 130 and 150 V for prodelphinidins) were used. The optimal collision energy for all transitions was 15 eV. Measured MRM peaks areas were used for calculation of mPD [25].

### 2.4. Determination of Antioxidant Activity

An accurate sample (0.05 g) was placed into a conical flask with 50 mL of 50% methanol, filtered through 0.22 µm filters and subjected to analysis. An ABTS∙+ radical cation decolourization assay was applied. An ABTS∙+ radical cation decolourization assay was applied according to the methodology described by [26]. A volume of 3 mL of ABTS∙+ solution (absorbance 1.00 ± 0.005) was mixed with 20 μL of the methanol extract of PSRE or PACN powder. A decrease in absorbance was at a wavelength of 734 nm after keeping the samples in the dark for 60 min. Antioxidant activity was expressed as Trolox equivalents (TE) in µmol/g DW of the extract.

The ferric reducing antioxidant power (FRAP) assay was applied according to the methodology described by Benzie et al. [27]. A volume of 3 mL of the prepared FRAP reagent (consisting of TPTZ (0.01 M in 0.04 M HCl), FeCl_3_·6H_2_O (0.02 M in water), and acetate buffer (0.3 M, pH 3.6) at the ratio of 1:1:10) was mixed with 20 μL of the methanol extract of PSRE or PACN powder. An increase in absorbance was recorded after 60 min at a wavelength of 593 nm. Antioxidant activity was expressed as Trolox equivalents (TE) in µmol/g DW of the extract.

### 2.5. Methods and Procedures Implicated for Antibacterial Activity Evaluation

#### 2.5.1. *Pelargonium sidoides* Root Extract (PSRE) Preparation

Two grams of root extract powder were resuspended in 10 mL of 60% methanol (Sigma, in ultra-pure water) and vortexed for 2 min to allow for a homogeneous powder dispersion. Then, the solution was incubated for 2 h at 50 °C with agitation in the dark. Afterwards, the tube containing powder solution was collected and centrifuged at 2000× *g* (at room temperature) for 20 min to pellet unsolved powder. Supernatant was collected, 0.45 µm filtered to remove debris, covered with tinfoil, and stored at 4 °C until use. To prepare freeze dried powder, the solution was first evaporated by speed vacuum (SCANVAC-Scan speed 32/40) under vacuum pressure (1.013 mbar) for 4 h; the final yield was approximately 50% (≈4 mL). Finally, concentrated solution was solved with 10 mL of ultra-pure water, stored overnight at −80 °C and then freeze-dried using a Scanvac machine (SCANVAC LABOGENE/COOL SAFE 55-4 with vacuum pump; T = −52 °C; P = 0.500 hpa) for 7 h. Obtained powder was stored at 4 °C until use for experiments; a fresh freeze-dried powder was prepared prior to each experiment. The powder was then solvated in ultra-pure water and filtered through a 0.22 μm filter prior to use on bacteria.

#### 2.5.2. Proanthocyanidins from Extract (PACN)

PACN extract was provided as freeze-dried powder. 0.05 g of PACN fraction powder were dissolved in 50 mL of ultra-pure water (Millipore, Vimodrone, Italy), vortexed for 2 min to allow complete dissolution and then filtered with a 0.22 µm filter. A fresh solution was prepared prior to each experiment and stored at 4 °C.

#### 2.5.3. Bacteria and Growth Conditions

All the bacteria used in the experiments were purchased from the Deutsche Sammlung von Mikroorganismen und Zellkulturen (*P. gingivalis* DSM 20709; *S. salivarius* DSM 20067; DSZM, GmbH, Braunschweig, Germany). Lyophilized bacteria were equilibrated following manufacturer’s instructions by using their specific media: brain heart infusion (BHI, Sigma Aldrich) supplemented with hemin and menadione (final concentration 5 mg/L and 0.5 mg/L, respectively) and tryptic soy agar (TSA) supplemented with 5% sheep blood for *P. gingivalis*; tryptic soy broth (TSB, Sigma Aldrich) and TSA plates for *S. salivarius*. Unlike the aerobic strains that were able to generate individual colonies after a single day of incubation at 37 °C, *P. gingivalis* was grown for 3 days at the same temperature in anaerobic conditions using Anaerobox (Thermo Fisher Scientific, Monza, Italy). Once round-single colonies were formed, fresh broth-cultures were prepared prior to each experiment by dissolving 2–3 colonies in 30 mL of each specific medium, as previously described. Bacteria concentration was adjusted until 1 × 105 cells/mL by diluting in fresh media until optical density of 0.001 at 600 nm was reached as determined by spectrophotometer (Victor, Packard Bell, Lainate, Italy).

#### 2.5.4. PSRE and PACN Antibacterial Activity

Starting from the mother solutions prepared as described previously, PSRE and PACN were tested for their antibacterial activity at different concentrations, as follows:

(i) PSRE water solution: 0.02, 0.03, 0.04, 0.05, 0.06, 0.07, 0.08 and 0.09 g/mL
(ii) PACN water solution: 0.01, 0.03, 0.05, 0.07 and 0.09 mg/mL

PSRE and PACN were individually mixed directly into each complete medium to obtain a final volume of 200 μL containing 1 × 10^5^ exponentially growing bacteria/mL into a 96 well-plate. Plates were then incubated at 37 °C for 2 days in anaerobic conditions for *P. gingivalis* and for 1 day in aerobiosis for *S. salivarius*, respectively. Anaerobic conditions were maintained for 2 days prior to test viability due to the longer growth time of *P. gingivalis*, as reported by the strain provider’s guideline (DSMZ). In this way, both the tested strains were allowed to produce biofilm in a comparable manner prior to undergo metabolic analysis. Bacteria viability was evaluated by the colorimetric Alamar blue assay (alamarBlue^®^, Thermo-Fisher, Waltham, MA, USA); briefly, the ready-to-use solution was added to each well in a 1:10 ratio and incubated for 4 h in the dark at 37 °C. After incubation, 100 μL were collected from each test specimens into 1.5 mL tubes, centrifuged to remove any residual debris and finally spotted to a new black-bottom 96-well plate. Fluorescence was recorded at 590 nm using a spectrophotometer. Wells containing only extract + Alamar blue were used as blanks while bacteria cultivated with each complete medium without the extract were considered as positive untreated controls and considered to have 100% viability.

#### 2.5.5. Statistical Analysis

The amounts of phenolic compounds were expressed as a mean ± standard deviation (SD) of three replicates. The statistical data analysis was evaluated by applying the ANOVA with Tukey HSD post hoc test. Differences were considered statistically significant when *p* < 0.05. The data was processed using Microsoft Office Excel 2010 (Microsoft, Redmond, WA, USA) and SPSS 20 (IBM, Armonk, NY, USA) software. For antibacterial efficiency evaluation, the experiments were performed in 16 replicates. Data were analysed by the SPSS software (v20, IBM) using the one-way ANOVA and the Sheffè tests as post hoc. Significance was set at *p* < 0.01.

## 3. Results

### 3.1. Composition of Phenolic Compounds of PACN from PSRE

For the identification of the composition of the phenolic compounds, PACN was separated from PSRE and collected as an acetone fraction (AF) by means of Sephadex LH-20. The freeze dried PACN preparation (from AF) yielded 1.37 ± 0.07 g and comprised approximately 34.25% of the loaded PSRE.

**Figure 1:**
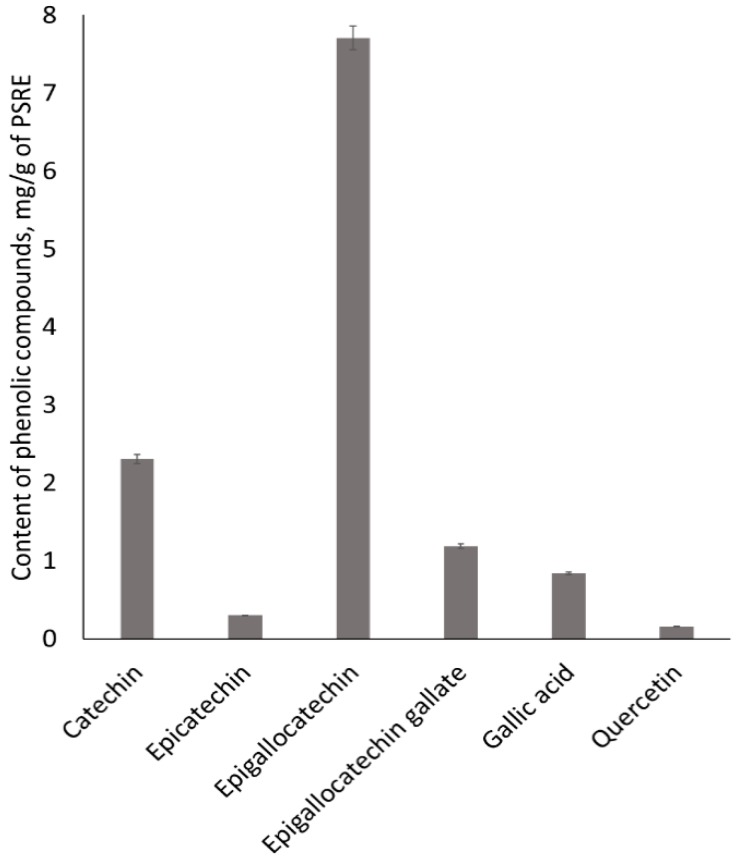
Content of phenolic compounds (mg/g) determined in *Pelargonium sidoides* root extract (PSRE).

Six phenolic compounds—catechin, epicatechin, epigallocatechin, epigallocatechin gallate, gallic acid, and quercetin—were identified and quantified in PSRE (Figure 1 and Figure S1).

Epigallocatechin (7.71 ± 0.15 mg/g, *p* < 0.05) was the predominant phenolic compound, and the amounts of other identified and quantified phenolic compounds were significantly lower. All identified phenolic compounds were detected in the methanol eluate after purification of the PSRE extract. The profile of the freeze-dried AF contained none of the abovementioned phenolic compounds and was comprised only of PACN.

PACN was characterized by different degrees of polymerization, and therefore the application of reverse phase HPLC is very complicated and a colorimetric assay based on butanol/HCL hydrolysis was employed [24,28]. After hydrolysis, the HPLC profiles of PSRE and a freeze-dried PACN sample revealed that all of the PACN can be ascribed to prodelphinidins as the peak detected in the chromatogram was that of delphinidin. The total amount of prodelphinidin (expressed as delphinidin equivalents) was 570.29 ± 9.49 mg/g, with 782.60 ± 6.50 mg/g and 9.87 ± 0.21 mg/g of PSRE, PACN and methanol eluate, respectively. The highest (*p* < 0.05) prodelphinidin content was found in PACN (Figure S2).

To present a more detailed view of the PACN composition, the PSRE and PACN aliquots were analysed using UPLC-MS/MS (data presented in Table 1).

ESI-MS full-scan analyses exhibited ions at *m*/*z* 609, 913, 1217, 1521, and 1825 for PACN, corresponding to the deprotonated molecular ions of prodelphinidin oligomers from dimer to hexamer. This is in agreement with the results of Schotz et al. The authors proposed that even higher oligomers could be present in the *Pelargonium sidoides* prodelphinidin fraction, but they could not be detected under ES-MS conditions [25]. Prodelphinidins are composed of epigallocatechin and gallocatechin subunits.

A UPLC-ESI-MS system was applied for the identification of phenolics. Components were identified according to their retention time and a mass fragmentation spectrum that was compared to literary data. Compounds 1–10 were found in PSRE, while PACN contained only compounds 6–10. Compound 1 represents a molecular ion [M − H]^−^ at *m*/*z* 441 with a fragment ion at *m*/*z* 289, which is a typical mass in the negative mode of epicatechin. The MS spectra indicate the existence of a galloyl group (152), and therefore compound 1 could be identified as epicatechin gallate. Compound 2 showed a molecular ion at *m*/*z* 457 and was identified as epigallocatechin-gallate due to the presence of fragment at *m*/*z* 305, as the result of a loss of galloyl moiety. Compounds 3 and 4 were identified as catechin and epicatechin, and their identities were confirmed by the standards.

Compound 5 showed an ion *m*/*z* 305 and was identified as epigallocatechin; the fragment ion at *m*/*z* 261.760 was due to the loss of CO_2_ [29,30]. Compounds 6–10 were identified as derivatives of prodelphinidins. The characteristic fragmentation pathways for PACN are the quinone methide mechanism, heterocyclic ring fission, and retro-Diels-Alder (RDA) fission with neutral losses of 168 Da [24]. These compounds produced *m*/*z* ions of 609, 913, 1217, 1521, 1825, respectively, with various fragment ions (Table 1) obtained by the loss of a galloyl and RDA fragments [30,31].

### 3.2. Determination of the Mean Degree of Polymerization of PACN

UPLC-MS/MS analysis was used for the estimation of the mean degree of polymerization (mDP) for the oligomers and polymers in Pelargonium sidoides root extract (Figures S3–S6). The determination of mDP is based on the mMRM quantification of both terminal and extension units of the most of the common PACN subclasses—e.g., procyanidins (*m*/*z* 287 and 289 respectively) and prodelphinidins (*m*/*z* 303 and 305 respectively)—according to a method described by Engstrom et al. [32]. The calculated mean degree of polymerization for the analysed PACN sample was 6.0.

### 3.3. Antioxidative Activity of PCANs and PSRE

Oxidative damage and oxidant-induced signalling is involved in numerous pathologies; therefore, it is important to look for new potent and biocompatible antioxidants. For this reason, we have evaluated the antioxidant activity of PACN isolated from PSRE and compared it with that of the PSRE solution. The antioxidative capacity was examined by free radical scavenging efficiency and reducing activity using a 2,2-azinobis (ethyl-2,3-dihydrobenzothiazoline-6-sulphonic acid) diammonium salt (ABTS) assay and ferric reducing antioxidant power (FRAP) assay, respectively. The antioxidant activity for PSRE and PACN were 3747.75 ± 534.56 µmol/g and 4472.75 ± 707.71 µmol/g of TE, respectively. The reducing activity for PSRE and PACN were 3210.17 ± 108.17 µmol/g and 4111.31 ± 150.74 µmol/g of TE, respectively. The PACN fraction consisting of prodelphinidin oligomers showed significantly (*p* < 0.05) higher Trolox equivalent radical scavenging and reducing activity compared to PSRE. This is in agreement with the results of other researchers who also determined the greatest antioxidant values for the prodelphinidin-containing fractions [31].

### 3.4. Antibacterial Activity of PSRE and PACNs

To test the antibacterial properties of PSRE and PACN isolated from the extract, the effect of a concentration range of PSRE and PACN solutions on the viability of a Gram-negative biofilm former periodontal/peri-implant keystone pathogen *P. gingivalis* and a Gram-positive aerobic commensal bacteria (*Streptococcus salivarius*) was evaluated by colorimetric Alamar blue assay. Bacteria cultivated with fresh untreated medium were used as control.

**Figure 2:**
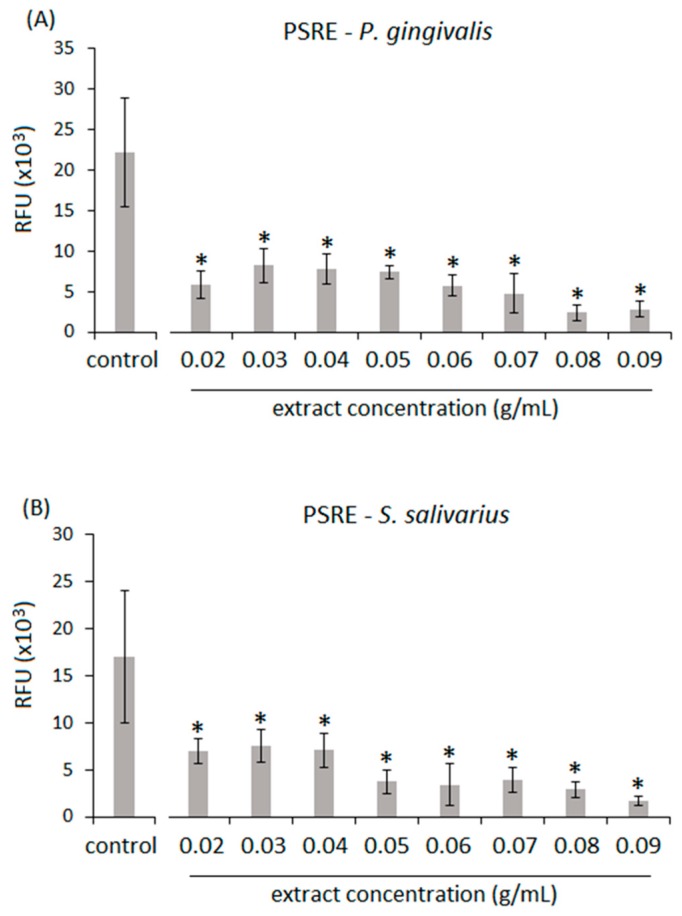
Antibacterial activity of *Pelargonium sidoides* root extract (PSRE). The extract at all tested concentrations (expressed as g/mL) induced a significant reduction of *P. gingivalis* (**A**) or *S. salivarius* (**B**) viability in comparison with the untreated control (*p* < 0.01, indicated by the *). Values are represented as the mean ± SD of 16 independent measurements.

Results related to PSRE antibacterial efficacy are reported in Figure 2. In general, PSRE extract was effective in reducing the viability of both *P. gingivalis* (Figure 2A) and *S. salivarius* (Figure 2B) in a significant manner in comparison to the untreated control starting from the lowest 0.02 g/mL concentration (a–b, *p* < 0.01, indicated by the *). Accordingly, all the tested concentrations demonstrated a high efficacy in reducing bacteria viability, resulting in a range between 12% and 36% for *P. gingivalis* and between 9% and 44% for *S. salivarius,* as summarized in Table 2. However, no differences were observed in the comparison of *P. gingivalis* and *S. salivarius* results; thus, despite its high efficacy, the PSRE extract did not show a selective activity towards pathogen or commensal strains.

**Figure 3:**
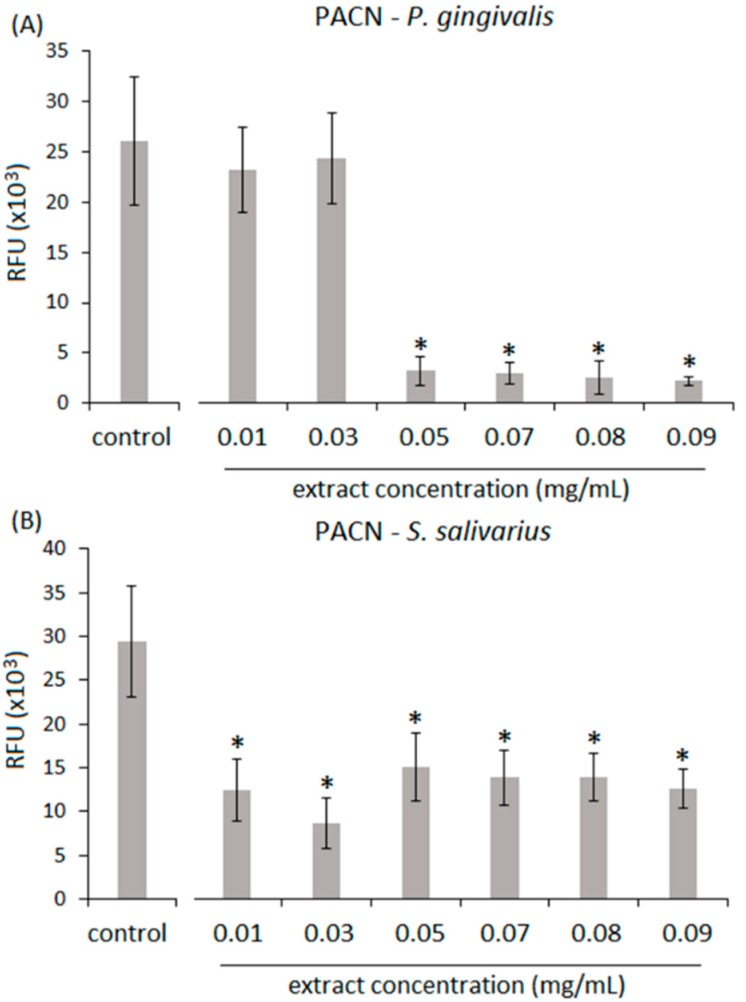
Antibacterial activity of proanthocyanidins (PACN). A different activity was noticed for various concentrations (expressed as mg/mL). For *P. gingivalis* (**A**) only concentrations >0.03 mg/mL were effective in significantly reducing viability (*p* < 0.01, indicated by the *) with results of approximately 10%. By contrast, PACN had moderate effect on (**B**) *S. salivarius* in comparison to *P. gingivalis*. Values are represented as the mean ± SD of 16 independent measurements.

Results related to PACN antibacterial efficacy are reported in Figure 3. As opposed to results obtained with the PSRE extract, PACN treatment resulted in different effects on viability between *P. gingivalis* and *S. salivarius*. In fact, the lower 0.01 and 0.03 mg/mL concentrations were not sufficient to determine a significant reduction of *P. gingivalis* viability in comparison with the control (Figure 3A, *p* > 0.05); by contrast, the same amount was effective in significantly decreasing *S. salivarius* viability (Figure 3B, *p* < 0.01, indicated by the *). However, when the concentrations were increased to 0.05–0.09 mg/mL, a strong decrease of *P. gingivalis* was observed (*p* < 0.01 in comparison with control, indicated by the *) while *S. salivarius* values were almost stable. Accordingly, considering the viability rate (Table 3), it was noted that the increase of PACN in solution reduced *P. gingivalis* viability from ≈90% (0.01–0.03 mg/mL) to ≈10% (0.05–0.09 mg/mL), while *S. salivarius* viability was always in the range between 40% and 50%. Thus, considering concentrations of 0.05–0.09 mg/mL, the activity of PACN extract was less effective against the commensal *S. salivarius* viability in comparison with *P. gingivalis*; in fact, even if the viability of the bacteria was significantly reduced in comparison to untreated controls, the values never fell below 39%. Thus, considering the previously mentioned concentrations of 0.05–0.09 mg/mL, PACN displayed a strong activity towards oral pathogen *P. gingivalis,* whose viability was reduced by about 90%, while a moderate effectiveness was obtained towards the commensal *S. salivarius,* whose viability was 50–60% reduced.

## 4. Discussion

The discovery of new infection treatment strategies in the race against increasing bacterial virulence and resistance is among the most important goals of contemporary science and healthcare. In addition, the strategy is not expected to destroy the part of human microbiota that positively contributes to skin, oral or intestinal physiology or the immune system. The constantly increasing average age of society in industrialized countries is turning the lack of efficient solutions in this field into a major medical problem. This challenge forces scientists and pharmacists to re-evaluate the significance of complex plant-derived antibacterial substances with mild yet multi-targeted activity. Multiple antibacterial properties of PSRE are well known in both folk and modern medicine and are usually attributed to the phenolic compounds of the herbal preparation. The literature indicates that the phenolic content of PSRE mainly comprises coumarins, phenolic acids, flavonols, flavan-3-ols and oligomeric PACN [10,33,34,35,36,37]. The increasing evidence about the strong antioxidant and anti-inflammatory yet immunity-stimulating antibacterial activity of PACN [37] suggests that the compounds could be potential replacements or supplements to antibiotic therapy. Our study was dedicated to the investigation of PACN from a PSRE preparation known for mild anti-infection efficiency and aimed to isolate, analyse and test the compounds for antioxidant and selective antibacterial action.

Because of the polymeric nature of PACN, the application of reverse phase HPLC is very complicated and the content of PACN was determined by colorimetric assay based on butanol/HCL hydrolysis. The experiments revealed that PACN isolated from PSRE was composed of prodelphinidins, the phenolic compounds characterized by 3,4,5-trihydroxyphenyl groups that are reported to be responsible for antioxidant and antibacterial activity [38,39,40].

Oxidant activity is implicated in the pathways of the etiopathogenesis of various diseases, such as cancer and cardiovascular, neurodegenerative, pulmonary, ocular disorders, and many others [41]. Thus, the search for efficient antioxidant compounds that could potentially be used for the prevention and treatment of these disorders or as a therapy-additive is of particular importance. Pereira et al. (2015) determined the superoxide, nitric oxide and peroxyl radical, DPPH scavenging and reducing activities of a *Pelargonium sidoides* commercial tincture [31]. The first candidates for antioxidant activity in PSRE are expected to be PACN, as there are numerous reports about the very potent antioxidant effects of these compounds extracted from different sources [42,43]. The significantly increased free radical scavenging activity of the PACN fraction compared to total PSRE preparation indicate that in pure PACN fraction, the activity is more concentrated and unmasked from other substances of PSRE. This result confirms that the compounds are suitable for the suppression of tissue-deteriorating inflammatory conditions. Additionally, besides the direct effect of preventing free radical-induced molecular injury, antioxidative effects are responsible for the induction of signal cascades supporting innate immunity, preventing cancerogenesis as well as autoimmune disorders [41,44]. Moreover, antioxidant activity can be related to tissue regenerating properties enhancing the anti-stress ability of human mesenchymal stem cells, stimulating intracellular self-renewal pathways and preventing cell senescence [41,45,46].

One of the most widely-spread infectious diseases that does not have an effective treatment strategy is periodontal disease. Periodontitis results from the unbalanced interaction between a subgingival biofilm and the host immune response [41,45]. Similarly, bacterial infection is considered to be the most important factor for implant failure [22]. Changes in biofilm composition are thought to disrupt homeostasis between the host and subgingival bacteria, thus resulting in periodontal/peri-implant tissue damage. In this scenario, some strains are strongly related to periodontal/peri-implant disease, whereas others are associated with healthy teeth and gums. Accordingly, it must be considered that, in the oral microenvironment, bacteria continuously interact with each other; therefore, the balance between the healthy and the diseased state should be considered to be a dynamic phenomenon where changes in ecology in the oral niche result in a shift towards the healthy or diseased state. Therefore, an ideal long-term antibacterial agent should be effective against putative pathogens and safe for commensals in order to preserve the balance. The most interesting discoveryof the study is that PACN at a certain concentration range exerts strain-selective antibacterial action, killing *P. gingivalis* much more efficiently than *S. salivarius*. This is a very important finding since *S. salivarius* is considered to be a beneficial bacterial strain [46], while *P. gingivalis* is recognized as a pathogen [19,47]. This strain-specific activity shown by the PACN preparation is very promising in terms of meeting the aim of selectively reducing pathogen viability within a complex microenvironment without compromising beneficial bacteria such as *S. salivarius*. To the best of our knowledge, this is the first case reporting such strain-selective activity of PACN. The antibacterial action of PCAN is attributed to biofilm disrupting properties by interfering with a N-acylhomoserine lactone-mediated quorum sensing of the bacteria [48,49]. PACN have also been shown to compromise adhesion to host cells by mimicking cell surface signalling [50] and lipopolysaccharide (LPS) binding [48]. LPS binding might lead to the destabilization of the outer membrane of the bacteria as reported in the study dedicated to the innate immunity protein lactoferrin action mechanism. Thus, LPS binding might be related not only to host protection against bacterial attachment and LPS-induced toxicity, but also to making the membranes of the pathogens more permeable and their inner systems more vulnerable. However, the exact mechanism of selective antibacterial activity of PACN from PSRE is yet to be determined, and this might provide a key to the new therapeutic strategy.

The most important feature of a good anti-infection therapeutic candidate is that it does not exert toxicity against the cells of the host. An in vivo study performed with rats revealed that PACN are non-toxic for gastric mucosal cells [51]. The antioxidant activity of the compounds, confirmed also in this study, should supposedly contribute to the reduction of inflammation and regeneration of the infected tissue. This adds to the value of PACN from PSRE as a possible therapeutic agent for the treatment of infections. However, more comprehensive studies on various cell and tissue types as well as prolonged treatment tests should be done to confirm the biocompatibility of PACN.

## 5. Conclusions

PACN from PSRE is a promising combination of natural antibiotics that selectively targets oral pathogenic microorganisms while preserving beneficial commensals. Considering that the mild and multi-target action of PACN is free from the risk of resistance development, the preparation is a good candidate for the development of prolonged treatment of oral disease as well as other infection strategies.

## Figures and Tables

**Table 1:** List of phenolic compounds identified in proanthocyanidins (PACN) fraction (A) and *Pelargonium sidoides* root extract (PSRE) methanol fraction (B).

**A**			
**PACN**	**Compound**	**[M − H]^−^*m*/*z***	**Product Ions *m*/*z***
6	Prodelphinidin dimer	609	305, 423, 441, 483, 565, 591
7	Prodelphinidin trimer	913	303, 423, 533, 483, 559
8	Prodelphinidin tetramer	1217	955, 1133, 1155, 1064, 1144, 1133, 732, 661
9	Prodelphinidin pentamer	1521	1421, 1283
10	Prodelphinidin hexamer	1825	609
**B**			
**PSRE**	**Compound**	**[M − H]^−^*m*/*z***	**Product Ions *m*/*z***
1	Epicatechin gallate	441	289
2	Epigallocatechin gallate	457	305
3	Catechin	289	
4	Epicatechin	289	
5	Epigallocatechin	305	179, 221, 261
6	Prodelphinidin dimer	609	305, 423, 441, 483, 565, 591
7	Prodelphinidin trimer	913	303, 423, 533, 483, 559
8	Prodelphinidin tetramer	1217	955, 1133, 1155, 1064, 1144, 1133, 732, 661
9	Prodelphinidin pentamer	1521	1421, 1283
10	Prodelphinidin hexamer	1825	609

**Table 2:** Viability rate of *P. gingivalis* and *S. salivarius* after treatment with *Pelargonium sidoides* root extract (PSRE). Assuming the control as having 100% viability, the survival rate of *P. gingivalis* resulted in a range between 12% and 36% (left panel) while *S. salivarius* was between 9% and 44% (right panel).

*P. gingivalis* Viability		*S. salivarius* Viability	
Extract (g/mL)	Viability (% vs. cnt)	Extract (g/mL)	Viability (% vs. cnt)
0.02	26.200	0.02	40.978
0.03	36.927	0.03	44.290
0.04	35.212	0.04	41.539
0.05	33.443	0.05	21.930
0.06	25.911	0.06	20.081
0.07	21.398	0.07	23.278
0.08	10.693	0.08	16.933
0.09	12.674	0.09	9.717

**Table 3:** Viability rate of *P. gingivalis* and *S. salivarius* after treatment with proanthocyanidins (PACN). Assuming the control as having 100% viability, the survival rate of *P. gingivalis* resulted in a range between 8% and 88% (left panel) while *S. salivarius* was between 39% and 51% (right panel).

*P. gingivalis* Viability		*S. salivarius* Viability	
Extract (mg/mL)	Viability (% vs. cnt)	Extract (mg/mL)	Viability (% vs. cnt)
0.01	88.919	0.01	42.345
0.03	93.379	0.03	39.408
0.05	12.299	0.05	51.330
0.07	11.436	0.07	47.193
0.08	9.612	0.08	47.156
0.09	8.488	0.09	42.964

## Supplementary Materials

The following are available online at http://www.mdpi.com/1996-1944/11/9/1499/s1, Figure S1: HPLC phenolic profile of methanol extract of Pelargonium sidoides root extract (λ = 280 nm), Figure S2: HPLC chromatogram of prodelphinidins hydrolysed using an n-butanol/HCl reagent (λ = 550 nm). Figure S3: Representative MRM chromatogram (transition 287 >> 125) of procyanidins extension unit at cone voltage 140 V, Figure S4: Representative MRM chromatogram (transition 289→245) of procyanidins terminal unit at cone voltage 140 V, Figure S5: Representative MRM chromatogram (transition 303→125) of prodelphinidins extension unit at cone voltage 150 V, Figure S6: Representative MRM chromatogram (transition 305→125) of prodelphinidins terminal unit at cone voltage 150 V.

## Abbreviations

PSRE	*Pelargonium sidoides* root extract
PACN	Proanthocyanidins
AF	Acetone fraction
LPS	Lipopolysaccharide
MRM	Multiple reaction monitoring
UPLC	Ultra-performance liquid chromatography
