# Supplementary material for: Efficacy of Proanthocyanidins from Pelargonium sidoides Root Extract in Reducing P. gingivalis Viability While Preserving Oral Commensal S. salivarius
Source: Materials (Basel). 2018 Aug 22;11(9):1499. doi: 10.3390/ma11091499 (PMC6164244; doi:10.3390/ma11091499)
Supplement: Supplementary file 1 [file materials-11-01499-s001.pdf]

Efficacy of proanthocyanidins from *Pelargonium sidoides* DC. root extract in reducing *P. gingivalis* periodontal pathogen viability preserving oral commensal *S. salivarius*

Nijole Savickiene [1,*], Aiste Jekabsone [2,3], Lina Raudone [1,2], Asmaa S. Abdelgeliel[4,5], Andrea Cochis[4], Lia Rimondini[4], Elina Makarova[6], Solveiga Grinberga[6], Osvalds Pugovics[6], Maija Dambrova[6], Ingrida M. Pacauskiene[7], Nomeda Basevičiene[7], Pranas Viškelis[8]

Supplementary Materials:

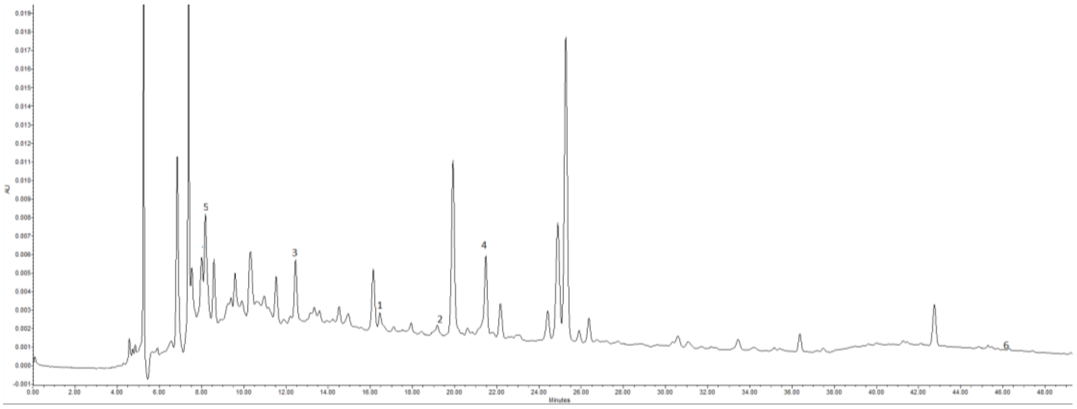

Figure S1.: HPLC phenolic profile of methanol extract of Pelargonium sidoides root extract (λ = 280 nm). Numbers indicate the peaks of analytes: (1) catechin, (2) epicatechin, (3) epigallocatechin, (4) epigallocatechin gallate, (5) gallic acid, (6) quercetin. HPLC system: Waters Alliance e2695 Separations Module equipped with a Waters 2998 PDA Detector (Milford, USA). The column: ACE Excel 3 SuperC18 analytical column (Aberdeen, Scotland) (250 × 4.6 mm, 3 μm) at 25∘ C. The mobile phase: 0.1% TFA in deionized water (A) and acetonitrile (B). The gradient: 0–30 min, 15%–30% B; 30–50 min, 30%– 60% B; 50–55 min, 60%–90% B; and 55–60 min, 90%–15% B. The flow rate was 0.5 mL min−1, and the injection volume was 10 μL.

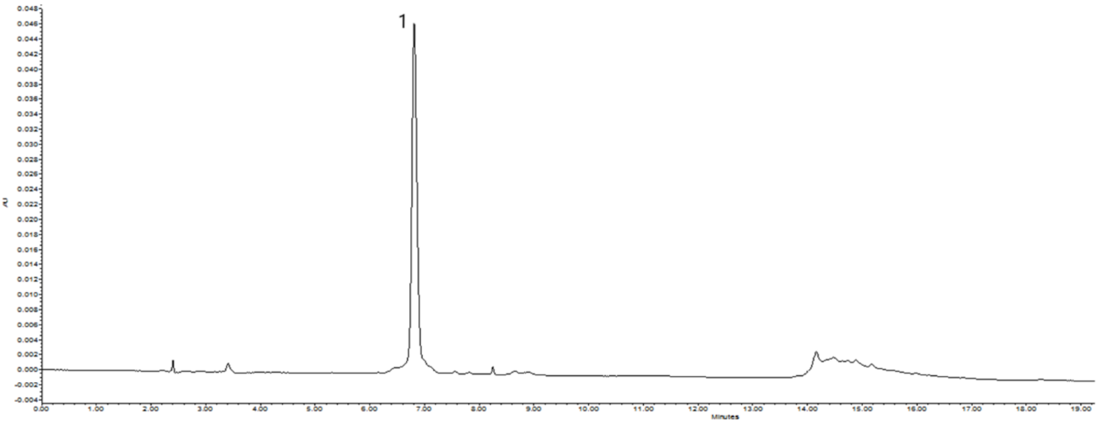

Figure S2.: HPLC chromatogram of prodelphinidins hydrolysed using an n-butanol/HCl reagent. (λ = 550 nm). Numbers indicate the peaks of analytes: (1) delphinidin. HPLC system: Waters Alliance e2695 Separations Module equipped with a Waters 2998 PDA Detector (Milford, USA). Column: ACE Excel 5 SuperC18 (250 × 4.6 mm, 5 μm) at 25∘ C. The mobile phase: 4% Phosphoric acid in deionized water (A) and acetonitrile (B). The gradient: 0–10 min, 15%–30% B; 10–15 min, 30%– 90% B; 15–17 min, 90%–90% B;

17–18 min, 90%–15% B; and 18-25 min, 15% B. The flow rate: 1 mL min−1, and the injection volume was 10 μL.

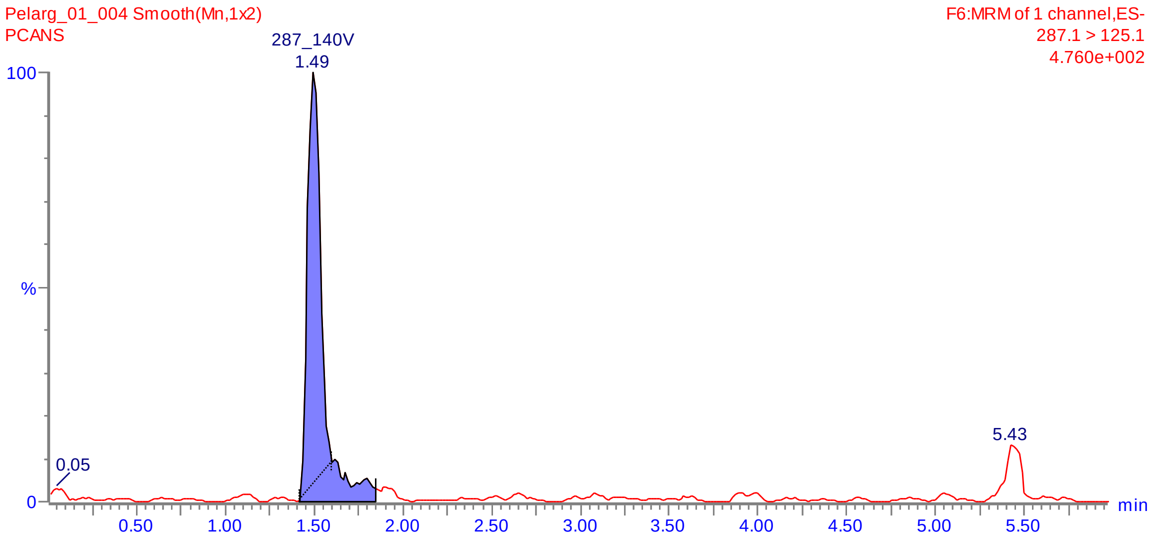

Figure S3. *Representative MRM chromatogram (transition 287>>125) of procyanidins extension unit at cone voltage 140V.* UPLC system: Acquity Waters connected with triple quadrupole mass spectrometer Quattro Micro Waters. Column: Acquity HSS T3 (2.1x50 mm, 1.8µm); Mobile phase: A: 0.1% Formic acid in water, B: Acetonitrile; Gradient: –Initial - 5%B, 0.5 min – 98%B , 4.5min -98%B, 4.7min -5%B, 6min – 5%B; Flow: 0.25mL/min; Column temperature: 30°C; Injection volume: 5µL. MS conditions: Ionization: ESI negative mode; Capillary voltage: 3.0kV; ESI source temperature: 150°C; Desolvation gas (N_2) flow: 800 L/h; Desolvation temperature: 400°C. Sample: 1mg/mL of PCANS in water.

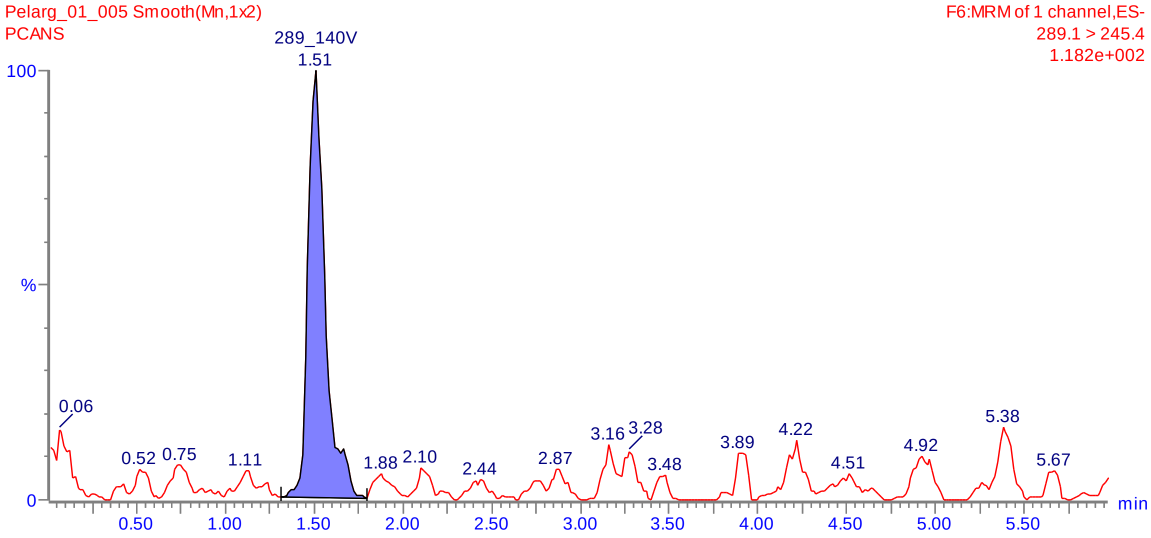

Figure S4. Representative MRM chromatogram (transition 289→245) of procyanidins terminal unit at cone voltage 140V. UPLC system: Acquity Waters connected with triple quadrupole mass spectrometer Quattro Micro Waters. Column: Acquity HSS T3 (2.1x50 mm, 1.8µm); Mobile phase: A: 0.1% Formic acid in water, B: Acetonitrile; Gradient: –Initial - 5%B, 0.5 min – 98%B , 4.5min -98%B, 4.7min -5%B, 6min – 5%B; Flow: 0.25mL/min; Column temperature: 30°C; Injection volume: 5µL. MS conditions: Ionization: ESI negative mode; Capillary voltage: 3.0kV;

ESI source temperature: 150°C; Desolvation gas (N2) flow: 800 L/h; Desolvation temperature: 400°C. Sample: 1mg/mL of PCANS in water.

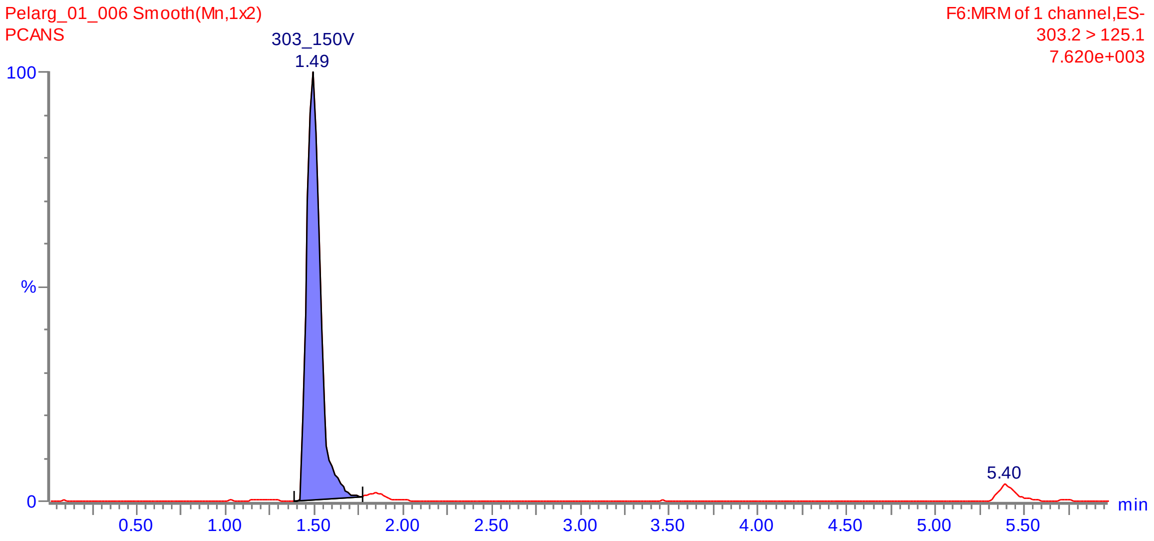

Figure S5. Representative MRM chromatogram (transition 303→125) of prodelphinidins extension unit at cone voltage 150V. UPLC system: Acquity Waters connected with triple quadrupole mass spectrometer Quattro Micro Waters. Column: Acquity HSS T3 (2.1x50 mm, 1.8μm); Mobile phase: A: 0.1% Formic acid in water, B: Acetonitrile; Gradient: –Initial - 5%B, 0.5 min – 98%B , 4.5min -98%B, 4.7min -5%B, 6min – 5%B; Flow: 0.25mL/min; Column temperature: 30°C; Injection volume: 5μL. MS conditions: Ionization: ESI negative mode; Capillary voltage: 3.0kV; ESI source temperature: 150°C; Desolvation gas (N2) flow: 800 L/h; Desolvation temperature: 400°C. Sample: 1mg/mL of PCANS in water.

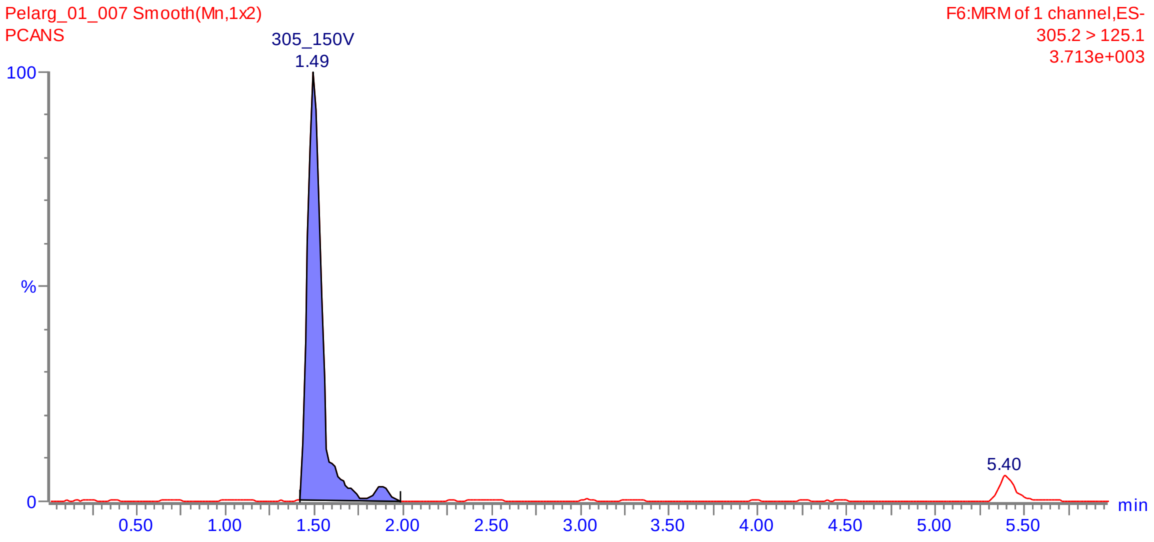

Figure S6. Representative MRM chromatogram (transition 305→125) of prodelphinidins terminal unit at cone voltage 150V. UPLC system: Acquity Waters connected with triple quadrupole mass spectrometer Quattro Micro Waters. Column: Acquity HSS T3 (2.1x50 mm, 1.8μm); Mobile phase: A: 0.1% Formic acid in water, B: Acetonitrile; Gradient: –Initial - 5%B, 0.5 min – 98%B , 4.5min -98%B, 4.7min -5%B, 6min – 5%B; Flow: 0.25mL/min; Column temperature: 30°C; Injection volume: 5μL. MS conditions: Ionization: ESI negative mode; Capillary voltage: 3.0kV; ESI source temperature: 150°C; Desolvation gas (N2) flow: 800 L/h; Desolvation temperature: 400°C. Sample: 1mg/mL of PCANS in water.
